# 1842676957299765Latent class cluster analysis to understand heterogeneity in prostate cancer treatment utilities

**DOI:** 10.1186/1472-6947-9-47

**Published:** 2009-11-27

**Authors:** Salimah H Meghani, Christopher S Lee, Alexandra L Hanlon, Deborah W Bruner

**Affiliations:** 1 Biobehavioral and Health Sciences Division; NewCourtland Center for Transitions and Health, University of Pennsylvania School of Nursing, Philadelphia, Pennsylvania 19104, USA; 2 Center for Bioethics, University of Pennsylvania, School of Medicine, Philadelphia, Pennsylvania 19104, USA; 3 Center for Health Outcomes and PharmacoEconomic Research, University of Arizona College of Pharmacy, Tucson, Arizona 85721, USA; 4 Recruitment, Retention and Outreach Core; Cancer Prevention and Control, Abramson Cancer Center, University of Pennsylvania, Philadelphia, Pennsylvania 19104, USA

## Abstract

**Background:**

Men with prostate cancer are often challenged to choose between conservative management and a range of available treatment options each carrying varying risks and benefits. The trade-offs are between an improved life-expectancy with treatment accompanied by important risks such as urinary incontinence and erectile dysfunction. Previous studies of preference elicitation for prostate cancer treatment have found considerable heterogeneity in individuals' preferences for health states given similar treatments and clinical risks.

**Methods:**

Using latent class mixture model (LCA), we first sought to understand if there are unique patterns of heterogeneity or subgroups of individuals based on their prostate cancer treatment utilities (calculated time trade-off utilities for various health states) and if such unique subgroups exist, what demographic and urological variables may predict membership in these subgroups.

**Results:**

The sample (N = 244) included men with prostate cancer (n = 188) and men at-risk for disease (n = 56). The sample was predominantly white (77%), with mean age of 60 years (SD ± 9.5). Most (85.9%) were married or living with a significant other. Using LCA, a three class solution yielded the best model evidenced by the smallest Bayesian Information Criterion (BIC), substantial reduction in BIC from a 2-class solution, and Lo-Mendell-Rubin significance of < .001. The three identified clusters were named *high-traders (n = 31)*, *low-traders (n = 116)*, and *no-traders (n = 97)*. *High-traders *were more likely to trade survival time associated with treatment to avoid potential risks of treatment. *Low-traders were *less likely to trade survival time and accepted risks of treatment. The *no-traders *were likely to make no trade-offs in any direction favouring the status quo. There was significant difference among the clusters in the importance of sexual activity (Pearson's χ^2 ^= 16.55, *P *= 0.002; Goodman and Kruskal tau = 0.039, *P *< 0.001). In multinomial logistic regression, the level of importance assigned to sexual activity remained an independent predictor of class membership. Age and prostate cancer/at-risk status were not significant factors in the multinomial model.

**Conclusion:**

Most existing utility work is undertaken focusing on how people choose *on average*. Distinct clusters of prostate cancer treatment utilities in our sample point to the need for further understanding of subgroups and need for tailored assessment and interventions.

## Background

The popularization of prostate-specific antigen (PSA) screening in the United States since the late 1980s has resulted in the early detection and sometimes over diagnoses of prostate cancer, which would not otherwise have been diagnosed within the patients' lifetime[1]. An increasing number of men are now challenged to choose between conservative management (watchful waiting) and a range of available treatment options including radical prostatectomy, radiation therapy, brachytherapy, and hormonal therapy, each carrying varying risks and benefits[2]. The trade-offs men face are between an improved life-expectancy with treatment accompanied by important risks such as urinary incontinence and erectile dysfunction. Since little consensus exists on which treatment will likely produce the best clinical outcomes,[3] the choices individuals make depend upon their personal appraisals of the net gains and losses associated with the treatment in question and the value one places on specific outcomes. Thus an explicit evaluation of patients' preferences using valuation techniques is increasingly reported in the medical decision-making literature.

Time trade-off (TTO) represents the most common scaling method used for assessing preferences for health states resulting from prostate cancer treatment[3,4]. TTO involves matching estimated years of remaining life with some health impairment to a shorter number of years in good health. TTO utilities are calculated as the ratio of time spent in full health to the time spent in a given health state (i.e., treated but with side effects). A TTO utility is a value between 0-1; where 0 signifies death and 1 represents perfect health. If a treatment outcome for erectile dysfunction carries a utility of 0.85, this means that each year lived is discounted by 15% for quality-of-life loss due to erectile dysfunction.

Previous studies of preference elicitation for prostate cancer treatment have found considerable heterogeneity in individuals' preferences for health states even given similar treatments and clinical risks. For instance, a recent meta-analysis reporting utilities for prostate cancer health states found that utilities for severe urinary problems ranged 0.48-0.96 and severe sexual problem ranged 0.61 to 1.0 across studies[4]. Thus patients with similar disease and overlapping clinical characteristics may demonstrate markedly different preferences for treatment outcomes[5].

Some factors have been shown to be associated with subjective preferences for treatment. For instance, age [6-8], ethnicity[7], marital status[9], education[9], current general health [7], and baseline sexual and urological functions[6-8,10] have been shown to be associated with prostate cancer utilities in previous studies. However, the findings remain inconsistent in predicting prostate cancer treatment utilities. Older men have been found to be less willing to accept the outcome of erectile dysfunction than younger men[6]. In another study, the willingness to trade off survival for sexual potency was significantly related to level of education and marital status but not age, race, income, current sexual or urinary function, or disease status[9]. Further, an integrated review of published studies on prostate cancer preferences revealed that many of the existing studies have been conducted with smaller samples and have employed at-risk patients only[3] representing trade-offs that may not be pertinent in a patient population. There remains a need to understand why subgroups of patients vary in their willingness to trade when presented with similar risks and uncertainties.

Latent class cluster analysis (LCA) is a relatively under-exploited technique in medical decision-making literature that can allow for the identification of unique subgroups based on their prostate cancer treatment utilities. LCA is a valuable method for theoretically important concepts that are not amenable to perfect measurement or direct observation. Treatment preferences associated with prostate cancer are a case in point. While we cannot directly observe prostate cancer treatment preferences we are likely to believe that individuals with certain type of preference structure will chose quality of life over survival gain or vice versa. LCA is based on the assumption that patterns of covariation among observed variables are due to each manifest variable's relationship to an unobserved latent variable[11]. If such a variable exists, then by studying the patterns of covariation among the observed variables, we can understand and characterize the nature of the latent variable[11].

Unlike traditional clustering (e.g., *k *means or fuzzy clustering approaches) that typically involves minimizing within- and maximizing between-cluster variance, cluster identification using LCA employs a model-based approach in which the 'probabilities' of class membership are estimated from model parameters and individuals' observed scores[12]. Individuals are assumed to belong to one of a set of *k *latent classes where the numbers and sizes of clusters are not known *a priori *[12]. LCA clustering approach allows simultaneous examination of several indicator (dependent) variables, such as utilities for several health states, as well as allows for the inclusion of covariates to identify predictors of class membership. Mixed measurement levels can be included in the model; thus no decisions ought to be made about scaling of the observed variables. No studies to our knowledge have employed this promising technique to understand heterogeneity in prostate cancer treatment utilities.

Using a two-step approach, we first sought to understand if there are unique subgroups of individuals based on their prostate cancer treatment utilities (calculated TTO utilities for various health states) and if such unique subgroups exist, then what demographic and urological variables may predict membership in these subgroups.

## Methods

### Sample and Setting

Data for this study was from a randomized controlled trial comparing TTO methodology with enhanced TTO (TTO+ visual analogue scale, VAS)[13,14]. The goal of the primary study was to establish the validity of enhanced TTO in improving consistency of utility elicitation (i.e., using the VAS to provide an anchor for the value placed on the worst possible outcome related to a particular health state, and referring to that anchor for all other trade-offs)[13,14]. The sample (N = 244) included men with prostate cancer (n = 188) and men at-risk (defined below) for disease (n = 56). Men with prostate cancer were receiving treatment at Fox Chase Cancer Center (FCCC), one of the National Cancer Institute designated comprehensive cancer centers located in the mid-Atlantic region. Men at-risk were recruited from the Prostate Cancer Risk Assessment Program (PRAP) established by FCCC in 1996 as a screening clinic and registry for the study of methods for early detection and factors that predispose to prostate cancer [15].

Eligibility for at-risk patients was similar to the criteria for entry into PRAP, i.e., any man between ages 35-69 years with at least one first degree relative or at least two second degree relatives on the same side of the family with the diagnosis of prostate cancer; African Americans with or without a first degree relative with prostate cancer; men who have tested positive for BRCA1. Ineligibility was based on inability to speak English and cognitive or emotional inability to understand the trade-offs to be made in the TTO interviews as determined by clinicians. Inclusion of men with prostate cancer included those with early stage disease treated at FCCC with curative intent with surgery or radiotherapy, and age was based on PRAP age criteria of 35-69 years.

Recruitment occurred through brochures and through local radio advertisements. Interviews occurred in the outpatient department of the FCCC and participants received $20 for the completion of the TTO interview. Informed and signed patient consents were obtained by trained research staff prior to any data collection. The study was approved by the Institutional Review Board of the Fox Chase Cancer Center.

### Measures

#### Demographics and Urological History

Demographic data were collected using an investigator-designed demographic form that collected information on age, race/ethnicity, education, income, marital status, and job status. Additional items elicited subjects' sexual and urological history, i.e., importance of sex for the subject (very important, somewhat important, unimportant), ability to have erection (usually able, sometimes a problem, usually a problem) and problems with urinary leakage (never a problem, sometimes a problem, usually a problem).

#### Health States and Utility Elicitation

Utilities were elicited using the TTO interview (n = 121) and the enhanced TTO interview (n = 123). Respondents were asked to express their preferences among common health states resulting from the treatment of localized prostate cancer treatment. There were 4 single states (40% risk of erectile dysfunction with radiation therapy, RT; 80% risk of erectile dysfunction with radical prostatectomy, RP; 10% risk of incontinence with RT; and 30% risk of incontinence with RP) and one joint state (40% risk of erectile dysfunction + 10% risk of incontinence with RT). Probabilities for risk of health states associated with therapies were derived from an extensive review of the literature at time (1997) and in consultation with an expert panel of urologists and radiation oncologists. Subjects were offered a choice between living a longer period of time in years with a side-effect versus living shorter periods of time without side-effects (watchful waiting). Survival or time without symptoms was presented in successively shorter increments of time until the subject was indifferent to the trade.

### Statistical Analyses

There were no statistically significant differences in utilities between experimental and control groups or between men with cancer or those at-risk for developing prostate cancer, thus data on all subjects were merged for this secondary analysis. The TTO utilities were calculated as:

Where U = predicted utility, X = time traded and Y = time horizon.

First, the skewed utility data were transformed into categories of utility. Trades based on the four single and one joint health state were transformed into three categories, i.e., utility between 0 to .49; .50 to .74; and .75 to .99. Consistent with the suggestion[16] that individuals who refuse to trade at all represent a unique group, we separately included a category of respondents who refused to trade at all (U = 1.0).

### Cluster Identification

Second, we performed a basic latent class mixture model analysis of utility categories using Mplus version 5.1 (Muthén & Muthén, Los Angeles, CA), employing the Lo-Mendell-Rubin adjusted test (LMR) [17], Bayesian Information Criterion (BIC) [18], and frequency to assess the performance of alternative models. The idea of "latent" class clusters is that it is not known what exactly is anchoring these clusters. Thus the statistical identification ofunique clusters using appropriate BIC and LMR/LRT criteria is significant in itself as it reveals any latent structure to the data. LMR assesses distribution of likelihood ratio test (LRT) for *k *and *k*-1 classes in evaluating appropriate number of clusters; a small *P*-value for LMR LRT suggests that the model with k classes is preferred over *k*-1 classes[17]. BIC takes into account the number of parameters used in model estimation[18] and rewards models with fewer classes that more accurately reproduce the data. Smaller BIC values are preferred as they represent model improvement over larger values.

#### Cluster Prediction

To predict cluster membership, we first evaluated utility classes for significant differences in demographics and urological history using two sample t-tests and ANOVA models to compare continuous variables and chi-square statistics to compare categorical variables (SPSS 15.0, Chicago, IL). Subjects' demographic and urological characteristics found significant at *P *≤ 0.2 on bivariate analysis were included in a multinomial logistic regression model to predict cluster membership (SPSS version 15.0, Chicago, IL). Model fit was determined using standard-likelihood ratio tests, while the impact of individual model factors was determined by evaluating log-likelihood ratio tests and calculating odds ratios (OR) and 95% confidence intervals (CI). A *P*-value of < 0.05 was predetermined as a cut-off for statistical significance.

## Results

The sample (Table 1) was predominantly white (77%), with a mean age of 60 years (SD ± 9.5). Most (77%) subjects had a diagnosis of prostate cancer, and most (86%) were married or living with a significant other. Calculated utilities and categories of utility are reported in Table 2. Mean utilities ranged from 0.84 (40% risk of erectile dysfunction + 10% risk of incontinence) to 0.95 (10% risk of incontinence). Unwillingness to make any trade-offs (TTO U = 1.0) was lowest for the joint state of 40% risk of erectile dysfunction+10% risk of incontinence (26.6%) and highest for 10% risk of incontinence (74.5%).

**Table 1:** Sample Descriptive Characteristics (n = 244)

Variable	Mean ± SD or n (%)
Age	60.36 ± 9.5
Self-Identified Race/Ethnicity:	
Caucasian	188 (77%)
African American	50 (20.5%)
Other	6 (2.5%)
Prostate Cancer:	
At-risk	56 (23%)
Active Disease	188 (77%)
Education: (n = 207)	
Less than High School	45 (21.7%)
More than High School	104 (50.2%)
Post Graduate	58 (28%)
Income: (n = 182)	
≤ $ 29,000/year	42 (23.1%)
$ 30,000, < $ 75,000/year	81 (44.5%)
≥ $ 75,000/year	59 (32.4%)
Marital Status: (n = 205)	
Married/Living with Sig. Other	176 (85.9%)
Not Married	29 (14.1%)
Job Status: (n = 200)	
Working	118 (59%)
Not Working	82 (41%)
Sex Important: (n = 243)	
Very Important	97 (39.9%)
Somewhat Important	114 (46.9%)
Unimportant	32 (13.2%)
Ability to Have an Erection: (n = 223)	
Usually Able	109 (48.9%)
Sometimes a Problem	44 (19.7%)
Usually a Problem	70 (31.4%)
Problems with Urinary Leaking: (n = 242)	
Never Have a Problem	179 (74%)
Sometimes Have a Problem	44 (18.2%)
Usually a Problem	19 (7.9%)

**Table 2:** Sample TTO Utilities/Categories of Utility

Utility	Mean (SD) (IQR) or n (%)
40% Chance of Erectile Dysfunction**	.9156 (.146) (.857 to 1.0)
No-trade	137 (56.4%)
.75 to .99	82 (33.7%)
.50 to .74	19 (7.8%)
Up to .49	5 (2.1%)
	
80% Chance of Erectile Dysfunction*	.8636 (.187) (.786 to 1.0)
No-trade	94 (38.7%)
.75 to .99	108 (44.4%)
.50 to .74	30 (12.3%)
Up to .49	11 (4.5%)
	
10% Chance of Incontinence**	.9544 (.120) (.929 to 1.0)
No-trade	181 (74.5%)
.75 to .99	52 (21.4%)
.50 to .74	7 (2.9%)
Up to .49	3 (1.2%)
	
30% Chance of Incontinence*	.8831 (.186) (.857 to 1.0)
No-trade	114 (47.7%)
.75 to .99	92 (38.5%)
.50 to .74	23 (9.6%)
Up to .49	10 (4.2%)
	
40% Chance of Erectile Dysfunction + 10% Chance of Incontinence**	.8358 (.196) (.757 to 1.0)
No-trade	65 (26.6%)
.75 to .99	133 (54.5%)
.50 to .74	32 (13.1%)
Up to .49	14 (5.7%)

TTO = time trade-off

** Risk associated with Radiation Therapy

*Risk associated with Radical Prostatectomy

### Cluster Identification using Latent Class Mixture Model

Using utility categories as indices of time trade-off, latent class mixture model analysis revealed several underlying distributions (Table 3). By evaluating both the LMR test and BIC several classes or clusters were identified as being informative. In our analysis, a three class solution yielded the best model evidenced by the smallest BIC value, substantial reduction (110.5) in BIC from the 2-class solution, and LMR significance of < .001 suggesting a statistically significant improvement in model fit. Thus, the three cluster solution was chosen for this analysis (Table 4).

**Table 3:** Latent Class Cluster Models of Prostate Cancer Treatment Utilities

Classes	LMR Test	*P*	BIC
2	320.7	< .0005	2266.2
**3**	**196.2**	**< .0001**	**2155.7**
4	59.7	.007	2183.3
5	53.7	.27	2216.8

BIC = Bayesian Information Criteria

LMR = Lo-Mendell-Rubin Adjusted Test

**Table 4:** Cluster Identification Using Latent Class Mixture Model Analysis

*Clusters*^†^	*High-traders n = 31* n (%)	*Low-traders n = 116* n (%)	*No-traders n = 97* n (%)
***Utilities***			

40% Chance of Erectile Dysfunction**			
No-trade	4 (12.90)	38 (32.76)	95 (97.94)
.75 to .99	5 (16.13)	76 (65.52)	1 (1.03)
.50 to .74	17(54.84)	2 (1.72)	0 (0.00)
Up to .49	5 (16.13)	0 (0.00)	0 (0.00)
			
80% Chance of Erectile Dysfunction*			
No-trade	0 (0.00)	17 (14.66)	77 (79.38)
.75 to .99	7 (22.58)	82 (70.69)	19 (19.58)
.50 to .74	13 (41.94)	17 (14.66)	0 (0.00)
Up to .49	11 (35.48)	0 (0.00)	0 (0.00)
			
10% Chance of Incontinence**			
No-trade	9 (29.03)	75 (64.66)	97 (100)
.75 to .99	11 (35.48)	41 (35.34)	0 (0.00)
.50 to .74	7 (22.58)	0 (0.00)	0 (0.00)
Up to .49	3 (9.68)	0 (0.00)	0 (0.00)
			
30% Chance of Incontinence*			
No-trade	2 (6.54)	22 (18.97)	90 (92.78)
.75 to .99	3 (9.68)	87 (75.00)	2 (2.06)
.50 to .74	16 (51.61)	4 (3.45)	3 (3.09)
Up to .49	9 (29.03)	1 (0.86)	0 (0.00)
			
40% Chance of Erectile Dysfunction + 10% Chance of Incontinence**			
No-trade	1 (3.23)	7 (6.03)	57 (58.76)
.75 to .99	1 (3.23)	101 (87.07)	31 (31.96)
.50 to .74	17 (54.84)	8 (6.90)	7 (7.22)
Up to .49	12 (38.71)	0 (0.00)	2 (2.06)

^†^*P *<.0001(based on Bayesian Information Criteria & Lo-Mendell-Rubin Adjusted Test (see Table 3)

** Risk associated with Radiation Therapy

*Risk associated with Radical Prostatectomy.

Based on the utility structures, the three clusters were named *high-traders*, *low-traders*, and *no-traders*. A small group (n = 31) was *high-traders*; individuals in this cluster were more likely to trade survival time that is associated with treatment to avoid potential risks of treatment. Majority (n = 116) were *low-traders*; individuals in this cluster were less likely to trade survival time associated with treatment and accepted potential risks of treatment. The *no-trade *cluster was more likely to have individuals who were unwilling to make trade-offs in any direction favouring watchful waiting or status quo (n = 97).

On bivariate analysis, the three clusters did not differ significantly in age, ethnicity, education, income, marital status, employment status, prostate cancer vs. risk of prostate cancer, ability to have an erection, or urinary leaking. There were, however, significant differences in the importance of sexual activity (Pearson's χ^2 ^= 16.55, *P *= 0.002; Goodman and Kruskal tau = 0.039, *P *< 0.001). That is, *high-traders *were more likely to find sex very important, *and low-traders *were more likely to find sex unimportant.

### Prediction of Cluster Membership

In combination, age, race/ethnicity, history of prostate cancer and the importance of sexual activity were significant in explaining likelihood of class membership (χ^2 ^= 25.7812, *P *= 0.004). These variables were included in a multinomial logistic regression model (Table 5). Race/ethnicity and level of importance assigned to sexual activity were independent predictors of class membership. Non-white subjects were less likely to be *low-traders *than *no-traders *(OR 0.409) and more likely to be *high-traders *than *low-traders *(OR 3.222). Subjects who indicated that sexual activity was very important were much more likely to be *high-traders *(OR 7.063) than *no-traders*, and *low-traders *than *no-traders *(OR 4.506). Subjects who indicated that "sexual activity was somewhat important" were also much more likely to be *low-traders *than *no-traders *(OR 4.377). Age and diagnosis of prostate cancer were not significant factors in the multinomial model.

**Table 5:** Multinomial Logistic Regression Results for Latent Classes of Prostate Cancer Utilities

Covariate	*High- vs. No-traders* OR (95% CI)	*Low- vs. No-traders* OR (95% CI)	*High- vs. Low-traders* OR (95% CI)
Age	1.046 (0.982 to 1.113)	1.021 (0.979 to 1.064)	1.025 (0.964 to 1.089)
Non-white White (reference)	1.319 (0.444 to 3.921)	0.409 (0.182 to 0.920)*	3.222 (1.071 to 9.694)*
Men with prostate cancer Men at-risk (reference)	0.787 (0.181 to 3.411)	0.460 (0.170 to 1.245)	1.711 (0.411 to 7.126)
Sex very important Sex unimportant (reference)	7.063 (1.413 to 35.301)*	4.506 (1.705 to 11.910)**	1.568 (0.291 to 8.443)
Sex somewhat important Sex unimportant (reference)	3.052 (0.607 to 15.337)	4.377 (1.745 to 10.981)**	0.697 (0.128 to 3.800)

*= *P *< 0.05; **= *P *< 0.01; CI = confidence interval; OR = odds ratio

### Cluster Membership Uncertainty

It is important to note that in latent class analysis, class membership is not determined with certainty for each individual; rather each individual has a probability of belonging to one of the latent classes. Thus class membership uncertainty should be taken into account in studying the association of patient characteristics with the latent classes [19]. Rigorous analytic techniques have been developed to account for bias that may result from moderate to substantial uncertainty derived from latent class analysis [19]. Based on our data, the median probability of being classified in class I (high-traders) for patients in class I was 1.000 with an inter-quartile range of .996 to 1.000. Eighty five percent of patients in this class had a probability of being in this class of .95 or above, and all but 2 had a probability of being in this class less than .80. The median probability of being classified in class II (low-traders) for patients in class II was .995 with an inter-quartile range of .968 to .999. Eighty five percent of patients in this class had a probability of being in this class of .95 or above and 10% had a probability less than .80. The median probability of being classified in class III (no-traders) for patients in class III was .999 with an inter-quartile range of .997 to 1.000. Eighty five percent of patients in this class had a probability of being in this class of .95 or above and 10% had a probability less than .80. Thus, with so few cases with moderate and none with substantial uncertainty with this particular 3 class solution, we believe there is limited bias derived from selecting the most likely cluster membership for each patient case and our methodological approach of studying the association of individual patient characteristics with these latent classes.

## Discussion

Previous studies have identified heterogeneity in treatment preferences related to prostate cancer. We used LCA to characterize heterogeneity previously reported to understand if there are unique subgroups of individuals based on their observed TTO utilities. LCA is a 'person-centered' rather than variable-centered approach to understanding heterogeneity within the data [20]. While based on aggregatedata onTTO utilitiespresented in Table 2, we know that there is heterogeneity in preferences but LCA offers little more insight about how subgroups of people make choices. For instance, clusters analysis (Table 4) tells us that heterogeneity is not based on 'risk' for '*high-traders' *and *'no-traders'*. Majority of the 31 people classified as *high-traders *traded high across health states regardless of amount (TTO utility) or type of risk (erectile dysfunction versus incontinence). Similarly, majority of the 97 people identified as *no-traders *were likely to choose status quo across risk categories regardless of type and amount of risk presented. This may mean that people in these two categories trade based on personal characteristics rather than risks presented, which raise questions about the theory driven hypothesis that people trade based on risks presented, i.e., people are risk-averse in the domain of gains and risk-seeking in the domain of losses [21].

Thus theheuristic clusters identified may aid in hypothesis generation such as, *high-traders *(those who trade life years to avoid risk of treatment), may be more risk averse, and may seek to enroll less in curative treatments or clinical trials with known or unknown risks. From a methodological standpoint, individuals in both *high-trade *and *no-trade *categories may respond less to interventionsdesigned to improve logical trade-offs. Further, from methodological standpoint, our findings of cluster identification also offer insights about what level of risk may be meaningfully interpreted by the participants while completing a TTO task. For instance, majority of individuals across the three clusters traded between *no-trade *to *low-trade *in the risk category of 10% chance of incontinence with radiation therapy. Our interpretation is that the risk is so marginal that participants may have had a hard time appraising it from the status quo.

With regard to prediction of cluster membership, in our analysis of a broad range of utilities of patients with prostate cancer and those at-risk, we find that sexual importance is a strong determinant of utilities in this patient population. That is, when compared to patients who found sex unimportant, patients who reported sexual activity as being very important were seven times as likely to trade life associated with treatment to avoid risks of treatment (erectile dysfunction and incontinence). Our strong finding is in contrast with Singer et al., who found that the reported willingness to trade-off survival for sexual potency was not related to interest in sex or frequency of sexual intercourse[22].

Second, although our sample was not ethnically diverse, patients who self-identified as non-whites were more likely to trade at the extremes, as *high-traders *or *no-traders*. These findings may represent within group heterogeneity and a methodological limitation since we combined African Americans and other minorities as non-whites. This necessitates careful interpretation of this finding. Nonetheless, ethnic variations have been found to influence preferences and outcomes associated with prostate cancer treatment. In a veterans population newly diagnosed with localized prostate cancer, Knight et al., found that even after adjusting for marital status, education level, and treatment, blacks reported increased difficulty with sexual interest at 3 and 12 months when compared with whites[23]. It is likely that race may moderate its effects on utilities through an interaction with sexual interest. The finding needs to be confirmed with lager samples of ethnically diverse populations.

While we accounted for participants' baseline sexual and urinary function, cluster typology may also be influenced by other contextual factors such as risk perceptions and beliefs about treatment outcomes [5,10]. Future studies of typology accounting for these important contextual factors are warranted. Future studies using LCA method may also undertake more robust analytic techniques that have been developed to identify class membership uncertainty [19]. Given that our three cluster solution indicated limited cluster membership uncertainty, we believe there is limited bias derived from our present methodological approach of studying the most likely cluster membership for each patient case.

## Conclusion

Our goal for this study was to understand appropriateness of LCA as a methodological approach in understanding unique patterns of heterogeneity in prostate cancer treatment utilities. Our study was limited in that variables included were those in the dataset collected for the primary study and did not include variables such as length and severity of disease, experience with treatment, or quality of life measures. Further, we did not contextualize trading types based on patients' perceptions about treatment efficacy and outcomes. Nevertheless, we demonstrated that there is a latent structure to prostate cancer treatment utilities. Most existing utility work is undertaken with the assumptions that groups under study represent a "single decision-making unit" [16](*p*.1073) with mean utilities representing preferences of individuals in that group. While the policy-level goal for cost-utility analysis has been to allocate resources based on how people chose on average, such approach stands in contrast with the current movement towards patient-reported outcomes focusing on patient-centered, tailored assessment and care. A consistent shift is needed in the conceptual and methodological approaches to move the decision science away from one-size-fits-all approach.

## Competing interests

The authors declare that they have no competing interests.

## Authors' contributions

This study was conceived by SHM and CSL using data from DWB's primary study. SHM and CSL contributed to all aspects of research and writing. DWB and ALH provided conceptual and methodological input during all the stages of research and manuscript development. Writing of the manuscript was led by SHM and all authors provided feedback and approved the final version.

## Pre-publication history

The pre-publication history for this paper can be accessed here:

http://www.biomedcentral.com/1472-6947/9/47/prepub
